# Impact of single nucleotide variants in estrogen genes on ovarian cancer risk: a systematic review and meta-analysis

**DOI:** 10.1530/EO-25-0007

**Published:** 2025-08-27

**Authors:** M P Aguiar, M O Gomes, L F Ananias, L F Silva-Martins, A P Espindula

**Affiliations:** ^1^ Health Science Program, Federal University of Triângulo Mineiro, Uberaba, Brazil; ^2^ Molecular Oncology Research Center, Barretos Cancer Hospital, Barretos, Brazil; ^3^ Ribeirão Preto Medical School, University of São Paulo, Ribeirao Preto, Brazil; ^4^ Hematological Research Laboratory, Federal University of Triângulo Mineiro, Uberaba, Brazil

**Keywords:** polymorphism, ovarian cancer, estrogen, genetic

## Abstract

Ovarian cancer research increasingly emphasizes the role of genetic factors, particularly estrogen, a key hormone also implicated in breast cancer. Hypotheses such as incessant ovulation and hormonal stimulation of ovarian epithelial cells support a hormonal etiology for ovarian cancer. This systematic review and meta-analysis evaluated the association between estrogen-related gene polymorphisms and ovarian cancer susceptibility. A comprehensive search was conducted in EMBASE, LILACS, PubMed, Scopus, Web of Science, and Google Scholar. Study selection, data extraction, and risk of bias assessment were performed independently by five reviewers. We included observational studies in humans, with no restrictions on language or publication date that investigated associations between estrogen-related gene polymorphisms and ovarian cancer occurrence or susceptibility. Exclusion criteria included studies not addressing the research question, those involving non-human subjects, secondary analyses, and genes unrelated to estrogen. Thirty studies met inclusion criteria. Meta-analysis showed that the A allele of rs4680 in the *COMT* gene (OR 0.85; 95% CI: 0.73–0.99; *P* = 0.0373) and the G allele of rs1695 in *GSTP1* (OR 0.69; 95% CI: 0.51–0.92; *P* = 0.012) were associated with reduced risk of ovarian cancer. In contrast, the C allele of rs743572 in *CYP17A1* (OR 1.54; 95% CI: 1.29–1.84; *P* < 0.0001) was associated with increased risk. Despite promising findings, the limited number of studies and population heterogeneity may impact the robustness and generalizability of the results. These findings suggest a possible role for these polymorphisms in ovarian cancer risk and highlight the need for further studies. This review was registered in PROSPERO (CRD42023464116).

## Introduction

The incidence of ovarian cancer has been the focus of extensive research, particularly regarding the influence of genetics on predisposition to this disease and the prognosis of affected individuals. Estrogen, a steroid hormone, plays a crucial role in the development and progression of breast cancer, either through the stimulation of cellular proliferation, genotoxic effects, or by inducing aneuploidy (Russo & Russo 2006). Building on these findings, several studies have extended this connection to other types of cancer, particularly ovarian cancer, with an emphasis on the association between polymorphisms in genes related to estrogen metabolism and susceptibility to the disease.

The hormonal etiology of epithelial ovarian cancer is underpinned by two primary, not mutually exclusive, hypotheses that reflect current understanding of the disease. The first hypothesis posits that ‘incessant ovulation’ contributes to cancer development through the repeated rupture of the ovarian surface epithelium, leading to the formation of stromal fissures and inclusion cysts. The second hypothesis, often referred to as the gonadotropic hypothesis, suggests that hormonal stimulation of ovarian epithelial cells, either on the ovarian surface or within inclusion cysts, predisposes individuals to cancer, with estrogen being well-established as a key stimulator in this pathway (Sellers *et al.* 2005, Da Silva *et al.* 2020).

The degree to which estrogen stimulates or attenuates certain pathological conditions can be primarily explained by genetics. Polymorphisms in genes responsible for estrogen catabolism, such as *CYP1B1*, can alter cellular levels of genotoxic catechol estrogens and antiangiogenic 2-methoxyestradiol, thereby influencing the risk of ovarian cancer development. A population-based case–control study involving African American and Caucasian women revealed that individuals carrying the *CYP1B1* Leu(432) allele were more predisposed to ovarian cancer (Holt *et al.* 2007).

It is crucial to consider that cancer predisposition, in general, can also be influenced by low-penetrance genetic polymorphisms, similar to what is observed in other common disorders such as diabetes or breast cancer (Sellers *et al.* 2005, Fasching *et al.* 2009). These polymorphisms may modify estrogen metabolism, leading to variations in cancer susceptibility. For instance, studies have indicated that the use of contraceptive hormones can alter the association of specific polymorphisms, such as rs1271572 and rs1256030, with ovarian cancer risk, with the association being stronger among women who have never used contraceptive hormones (Lurie *et al.* 2009).

Other studies focus on polymorphisms in genes involved in estrogen synthesis, such as *CYP17A1*, *CYP19A1*, and *HSD17B1*, suggesting that genetic variations may influence ovarian cancer development (Kumar *et al.* 2022). The hypothesis posits that low-penetrance polymorphisms in genes involved in estrogen metabolism may play a role in the initiation of carcinogenesis (Delort *et al.* 2008). Furthermore, imbalances in estrogen metabolism can lead to the formation of estrogen-DNA adducts, which generate mutations in critical genes within ovarian epithelial cells (Cavalieri & Rogan 2016).

The current scientific literature suggests a strong association between estrogen, from its synthesis to its action, and ovarian cancer predisposition. Given the increasing use of genetics as a tool to understand estrogen’s role in this type of cancer, a systematic review of the literature was warranted. This review enabled a better understanding of the genes and polymorphisms involved, identified those associated with disease predisposition and prognosis, and highlighted unexplored genes that require further investigation. These insights could be integrated into diagnostic practice, supporting the use of genetic panels and preventive strategies.

## Methods

### Study design and protocol registration

This study was submitted and registered in the International Prospective Register of Systematic Reviews (PROSPERO) under registration number CRD42023464116.

### Search strategy and inclusion/exclusion criteria

The databases utilized for this systematic review included EMBASE, LILACS, PubMed, Scopus, and Web of Science, as well as the academic search engine Google Scholar. In addition, a manual search of the reference lists of all included studies was conducted. Articles selected for inclusion were required to contain the following keywords: ‘estrogen’, ‘polymorphism’, and ‘ovarian cancer’. The specific search strategies for each database are detailed in Supplementary Appendix A (see section on Supplementary materials given at the end of the article).

Observational studies conducted in humans, without language or date restrictions, published up until March 2025, and investigating the association of estrogen-related gene polymorphisms with the occurrence or susceptibility of ovarian cancer were included. The exclusion criteria were as follows: i) does not address the research question of the systematic review; ii) non-human population; iii) study derived from other research; iv) gene not related to estrogen.

Each single nucleotide polymorphism (SNP) in the included studies was evaluated for compliance with Hardy-Weinberg Equilibrium (HWE) in the control group, using a significance threshold of *P* > 0.05. Only SNPs with genotype distributions in equilibrium were included in the meta-analysis (Supplementary Appendix B).

### Research question

Is there an association between SNPs in estrogen-related genes and ovarian cancer? Adopted strategy: PECOS (participants, exposure, comparator, outcome, and study type):

P (participants): women diagnosed with ovarian cancer.
E (exposure): presence of SNPs in estrogen-related genes.
C (comparator): absence of SNPs in estrogen-related genes.
O (outcome): identification of associations between susceptibility and/or severity in relation to the presence of these polymorphisms.
S (study type): observational studies.

### Study selection

Reviewer calibration was conducted using the kappa index until a moderate level of agreement was achieved among the reviewers. Four independent reviewers (L F A, L F S M, M O G, and M P A) selected the included articles through a two-phase process. In phase 1, the four reviewers assessed the titles and abstracts according to the eligibility criteria. In phase 2, they reviewed the full texts and selected articles using the same criteria as in phase 1, subsequently cross-referencing all gathered information. In cases of discrepancies, a fifth reviewer (A P E) was involved to reach a final decision in both phases. The reference manager Rayyan was used in both phases, while EndNote Web was employed in the first phase to remove duplicate articles.

### Data extraction

Four independent reviewers (L F A, L F S M, M O G, and M P A) extracted data from the selected articles. Once the data were collected, they cross-verified the retrieved information with a fifth reviewer (A P E). The gathered information included author, study type, year of publication, country, patient characteristics (sample size and age), genotyping method, and the genes and polymorphisms studied (Table 1). In addition, the full genotype distributions (number of individuals for each genotype in cases and controls) for all polymorphisms included in the meta-analysis are provided in Supplementary Appendix C (genotype frequency tables).

**Table 1:** Characteristics of the studies included in the meta-analysis.

Authors/(Reference)	Year	Country	Study design	Cases	Controls	Ethnicity (case/control)	Gene	SNP	Genotyping method
Abaoğlu* et al.*	2021	Turkey	Case-control	47	47	Turkish population, ethnicity not specified	*COMT*	rs4860	TaqMan
Oliveira *et al.*	2012	Brazil	Case-control	132	132	Caucasian (126/126) Black (6/6)	*GSTM1 * *GSTT1 * *GSTP1*	Ile105Val	Multiplex PCR
Leite 2009	2009	Brazil	Case-control	383	423	White (84/203) Non-white (19/77)	*CYP17 * *CYP1A1 * *PRh * *ESR1* *COMT*	rs743572 rs4646903 rs1048943 PROGINS rs2234693 rs9340799 rs9322331 rs1784705 rs4680	PCR-RFLP
Dos Santos *et al.*	2017	Brazil	Case-control	24	61	Ethnicity not specified	*CYP17 * *GSTP1*	rs743572 Ile105Val	PCR-RFLP
Lurie *et al.* 2009	2009	USA	Case-control	313	574	Caucasians (72/148) Japanese (94/173) Hawaiians (69/106) Filipinos (36/79) Other Asian (17/27) Other (25/41)	*ESR2*	rs1271572 rs1256030 rs1256031 rs3020450	TaqMan allelic discrimination assay
Garner *et al.*	2002	USA	Case-control	563	523	Majority Caucasian	*CYP17 * *COMT*	rs743572 rs4680	PCR-RFLP
Goodman *et al.*	2000	USA	Case-control	108	124	Caucasian	*COMT*	rs4680	PCR-RFLP
Goodman. *et al.*	2001	USA	Case-control	129	144	Caucasians (36/42) Asians (58/69) Others (35/33)	*CYP1A1 * *CYP1A2 * *CYP1B1 * *COMT*	rs4646903 rs1048943 rs1056836 rs4680 rs762551	PCR-RFLP
Goodman *et al.*	2008	USA	Case-control	367^a^	602	Caucasians (90/158) Japanese (102/175) Hawaiians (82/119) Filipinos (43/80) Other (40/70)	*CYP19A1*	rs749292 rs727479	TaqMan allelic discrimination assay
Kumar *et al.* 2022	2022	India	Case-control	200	200	Women from Southern India, ethnicity not specified	*CYP17A1 CYP19A1 HSD17B1*	rs743572 rs10046 rs605056	TaqMan allelic discrimination assay
Gulyaeva* et al.*	2008	Russia	Case-control	96	180	Women from Novosibirsk, ethnicity not specified	*CYP1A1 * *CYP19 * *SULT1A1*	rs762551 rs700519 rs1042028	TaqMan
Heubner *et al.*	2010	Germany	Case-control	111	119	Ethnicity not specified	*CYP1A1*	rs1048943	PCR-RFLP
Matei *et al.* 2012	2012	Romania	Case-control	21	21	Ethnicity not specified	*CYP1A1 * *p53*	Códon 72/p53 polymorphism	PCR-RFLP
								rs1048943	
Mikhailova *et al.*	2006	Russia	Case-control	168	172	Ethnicity not specified	*CYP1A1 * *CYP1A2 * *CYP19 * *SULT1A1*	rs4646903 rs762551 Arg213His T264-C C734-A Arg264Cys G638-A	PCR-RFLP
Seremak-Mrozikiewicz *et al.*	2005	Poland and Germany	Case-control	110	212	Caucasians	*CYP1A1*	rs4646903 rs1048943	PCR
Pemmaraju *et al.*	2018	India	Case-control	80	100	Ethnicity not specified	*ESR1*	rs2234693	PCR-RFLP
Holt *et al.* 2007	2007	USA	Case-control	310	577	White (277/450) Black (33/127)	*CYP1A1*	rs464690 rs1048943	TaqMan
Schüler *et al.*	2014	Poland and Germany	Case-control	184	184	Caucasians	*ESR2*	rs3020450	Tetra-primer ARMS PCR
Sellers *et al.*	2005	USA	Case-control	503	609	Caucasians	*CYP1A1 * *CYP1B1 * *CYP1B1 * *CYP1B1*	rs1048943 rs10012 rs1056827 rs1056836 rs4680 rs9282861	PCR e Nanogen chip-based platform
Soltész *et al.*	2019	Hungary	Case-control	86	102	Caucasians	*MIR193B*	rs30236	PCR
Sugawara *et al.*	2003	Japan	Case-control	159	31	Japanese population, ethnicity not specified	*CYP1A1*	rs4646903 rs1048943	PCR and electrophoresis
Wu *et al.*	2005	Japan	Case-control	76	19	Japanese population, ethnicity not specified	*ESR1*	rs2234693	Direct sequencing PCR
Yazici *et al.*	2006	Turkey	Case-control	91	194	Turkish population, ethnicity not specified	*CYP17A1*	rs743572	PCR
Zahid *et al.*	2014	USA	Case-control	33	34	White (29/33) African American (4/1)	*CYP1B1 * *COMT*	rs1056836 rs4680	MALDI-TOF MS (matrix-assisted laser desorption/ionization time-of-flight mass spectrometry)

^a^ Mismatch between reported total number of participants and sum across ethnic groups.

### Risk of bias analysis

Given that the studies are observational, the Joanna Briggs Institute Critical Appraisal Checklist and the RevMan tool were utilized for the risk of bias assessment. The questions from the Joanna Briggs Institute checklist were input into the RevMan tool, which then generated a bar chart displaying the risk of bias for the included studies (https://revman.cochrane.org/info). The checklist comprised ten questions, with possible answers being: YES: low risk of bias (green color); NO: high risk of bias (red color); UNCLEAR: uncertain risk of bias (yellow color).

### Assessment of publication bias

To assess the presence of publication bias in the meta-analysis, both funnel plots and Egger’s regression test were employed. The analyses were conducted using the Metafor package in the R programming language (Viechtbauer 2010; https://www.r-project.org/). Funnel plots were used as a graphical method to visually inspect potential asymmetry, which may suggest publication bias. In addition, Egger’s regression test was applied as a statistical method to quantify the degree of asymmetry in the funnel plot by evaluating the relationship between the standard error and effect size. In this approach, a *P*-value lower than 0.05 in Egger’s test indicates the presence of publication bias.

### Data synthesis and statistical analysis

Heterogeneity among the studies was assessed using the *I*^2^ statistic, which describes the percentage of total variability attributed to actual differences between studies rather than to chance. Considering this heterogeneity, a random-effects model was used to perform the meta-analysis, allowing the incorporation of variability between study effects. In addition, prediction intervals were calculated to provide an estimate of the range in which the true effect of a future study is likely to fall, reflecting the heterogeneity observed among the included studies. To estimate the association of polymorphisms with ovarian cancer risk, odds ratios (ORs) with 95% confidence intervals were calculated. The significance level was set at 0.05. All analyses were conducted using the open-access R software (version 4.0.1).

In addition to the allele-contrast model, we also assessed dominant and recessive genetic models for each included SNP. In the dominant model, individuals carrying at least one copy of the variant allele (heterozygotes and homozygotes for the variant) are compared to those with two copies of the reference allele. In the recessive model, individuals with two copies of the variant allele are compared to those with at least one copy of the reference allele. The meta-analyses for these models were performed using Python, employing the statsmodels and metafor-py libraries for logistic regression and random-effects modeling. The results of these analyses are showed in Supplementary Appendix D.

The GRADE method (Grades of Recommendation, Assessment, Development and Evaluation Working Group) was utilized to assess the certainty of the evidence, determining how confident this systematic review is that the final estimate is close to reality. The GRADE approach considered factors such as risk of bias, indirect evidence, inconsistency of results, imprecision of estimates, publication bias, effect magnitude, and potential confounding factors. Summary tables of the findings were generated using the GRADEpro online software (Schünemann *et al.* 2008).

This systematic review was guided by the PRISMA checklist (Supplementary Appendices E and F) (Page *et al.* 2021).

## Results

**Figure 1:**
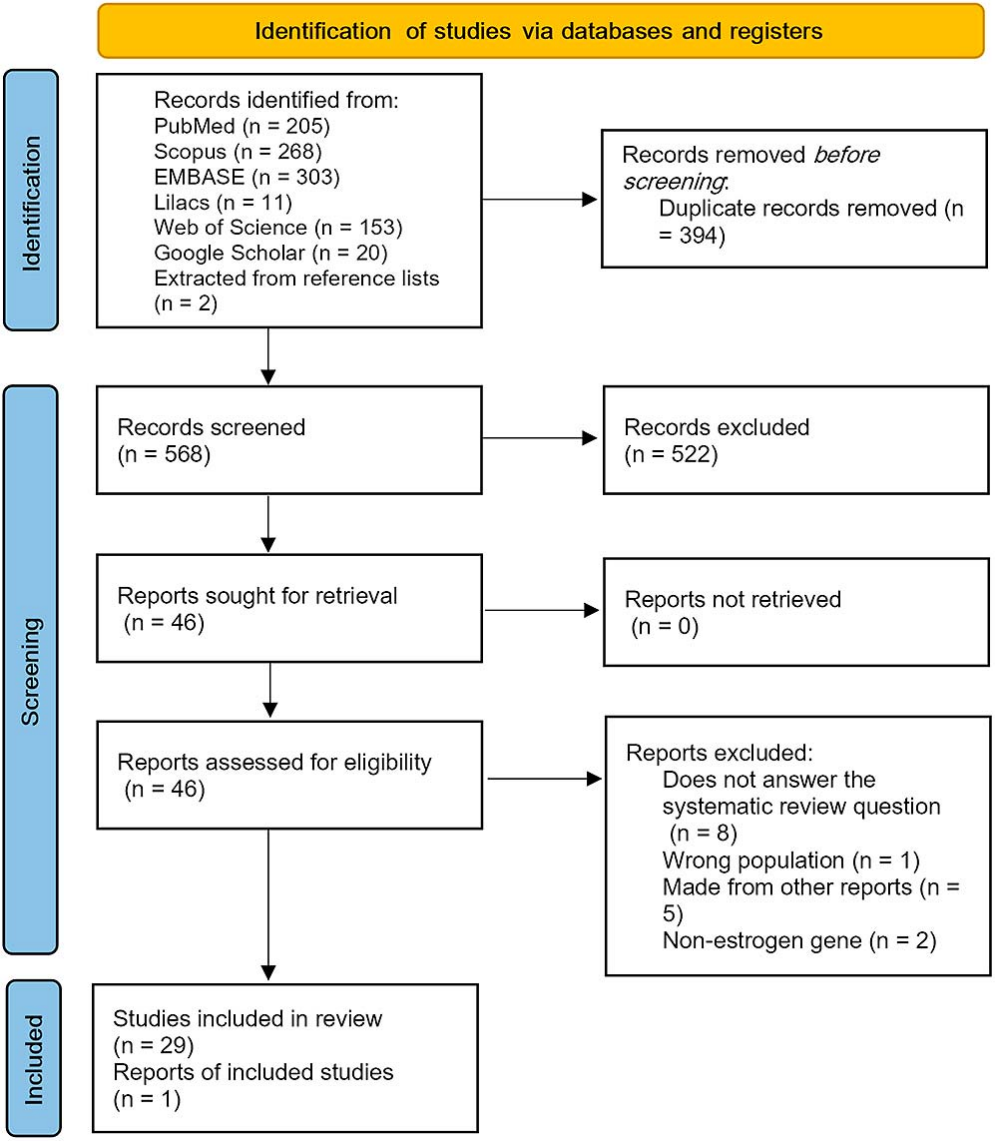
Flowchart illustrating the search strategy used to identify studies on the association between genetic polymorphisms and ovarian cancer. Source: prepared by the authors, 2025; adapted from PRISMA (Page *et al.* 2021).

In the initial calibration of the reviewers, we achieved a reasonable level of agreement (Kappa index = 0.21–0.8). After refining and better categorizing the eligibility criteria, we obtained moderate to substantial agreement among the reviewers (Kappa index = 0.41–1). A total of 1,032 studies were identified following the initial search across PubMed, Embase, Scopus, LILACS, Web of Science, and Google Scholar. As shown in Fig. 1, the database and gray literature searches yielded 1,032 articles. Of these, 416 were duplicates, and after their removal, 616 articles remained. Upon reviewing the titles and abstracts, 570 records were excluded for not aligning with the study’s theme, leaving 46 articles selected for full-text review. Following the full-text evaluation, 29 articles met the inclusion criteria. The articles excluded after full-text review, along with the reasons for their exclusion, are detailed in Supplementary Appendix G.

### Characteristics of included studies

A summary of the characteristics of the included studies can be found in Table 1, and the characteristics of the included SNPs can be found in Supplementary Appendix H.

### Risk of bias analysis

**Figure 2:**
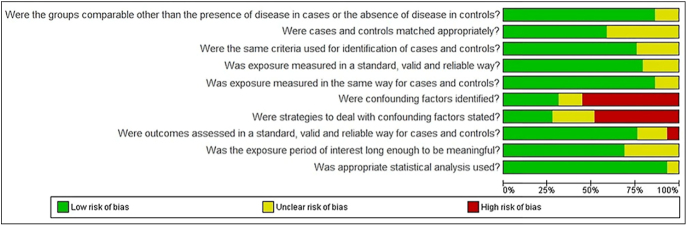
Risk of bias analysis for the included studies. Source: prepared by the authors, 2025; based on the RevMan tool (https://revman.cochrane.org/info). Green, low risk of bias; red, high risk of bias; yellow, uncertain risk of bias.

For most questions, the included studies exhibited a moderate to low risk of bias, with two studies presenting a moderate to high risk of bias (4.20) (Fig. 2). Conversely, six articles met all the criteria for methodological quality and were therefore considered to have a low risk of bias. The primary methodological limitation identified in approximately 69 and 72% of the studies was related to the identification of confounding factors and strategies for addressing them, respectively, as these elements were not well defined.

### Publication bias assessment results

The assessment of publication bias was conducted using funnel plots and Egger’s regression test for all included genetic variants. Among the analyzed variants, only one polymorphism, *CYP1A2* (rs762551), demonstrated a *P*-value less than 0.05 (*P* = 0.0021), suggesting evidence of publication bias. This result was derived from Egger’s regression test, which evaluates the relationship between standard error and effect size to detect asymmetry in the funnel plot.

For the other variants, the *P*-values were all above the 0.05 threshold, indicating no significant evidence of publication bias. All statistical details and corresponding funnel plots for each analyzed polymorphism are available in the supplementary materials for further inspection (Supplementary Appendix I).

### Meta-analysis results

#### *COMT*_rs4680 (NM_000754.4(*COMT*):c.472G>A (p.Val158Met))

**Figure 3:**
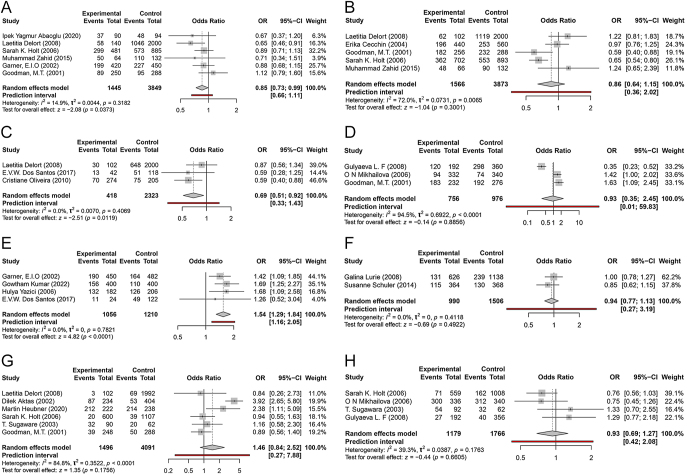
Forest plots of the polymorphisms in the COMT, CYP1B1, GSTP1, CYP1A2, CYP17, ESR2, and CYP1A1 genes. (A) COMT (rs4680): allele A; (B) CYP1B1 (rs1056836): allele G; (C) GSTP1 (rs1695): allele G; (D) CYP1A2 (rs762551): allele A; (E) CYP17 (rs743572): allele C; (F) ESR2 (rs3020450): allele A; (G) CYP1A1 (rs1048943): allele G; (H) CYP1A1 (rs4646903): allele C.

Six studies evaluated the rs4680 variant in the COMT gene, which were included in the meta-analysis of this work (Fig. 3A). An association was observed when the random-effects model was applied (OR = 0.85; 95% CI: 0.73–0.99), with low heterogeneity (*I*^2^ = 14.9%). The test for overall effect yielded *z* = −2.08 (*P* = 0.0373). The between-study variance was *τ*^2^ = 0.0044, and the prediction interval ranged from 0.66 to 1.11. The G allele was considered the reference in the meta-analysis.

#### *CYP1B1*_rs1056836 (NM_000104.4(*CYP1B1*):c.432C>G (p.Val432Leu))

The rs1056836 polymorphism in the *CYP1B1* gene was considered in five studies, which were subjected to meta-analysis. No significant associations were observed using the random-effects model (OR = 0.86; 95% CI: 0.64–1.15) (Fig. 3B). The heterogeneity among these studies was high (*I*^2^ = 72%). The test for overall effect yielded *z* = −1.04 (*P* = 0.3001). The between-study variance was *τ*^2^ = 0.0731, and the prediction interval ranged from 0.36 to 2.02. The C allele was considered the reference in the meta-analysis.

#### *GSTP1*_rs1695 (NM_000852.3:(*GSTP1*):c.313A>G (p.Ile105Val))

Three studies were included, and significant associations were observed when the random-effects model was applied (OR = 0.69; 95% CI: 0.51–0.92) (Fig. 3C). An *I*^2^ of 0% indicates no observed heterogeneity among the analyzed studies. The test for overall effect yielded *z* = −2.51 (*P* = 0.0119). The between-study variance was *τ*^2^ = 0.0070, and the prediction interval ranged from 0.33 to 1.43. The A allele was considered the reference in the meta-analysis.

#### *CYP1A2*_rs762551 (NM_000761.5:(*CYP1A2*):c.−163C>A)

The rs762551 polymorphism in the CYP1A2 gene was studied in three works, which were included in the meta-analysis. No significant associations were observed using the random-effects model (OR = 0.93; 95% CI: 0.35–2.45) (Fig. 3D). An *I*^2^ of 94.5% indicates high heterogeneity among the studies. The test for overall effect yielded *z* = −0.14 (*P* = 0.8856). The between-study variance was *τ*^2^ = 0.6922, and the prediction interval ranged from 0.01 to 59.83. The C allele was considered the reference in the meta-analysis.

#### *CYP17*_rs743572 (NM_000102.4(*CYP17A1*):c.−34T>C)

Four studies that considered the rs743572 polymorphism in the *CYP17* gene were included in the meta-analysis. After applying the random-effects model, significant associations were observed (OR 1.54; 95% CI 1.29–1.84) (Fig. 3E). The heterogeneity among the studies was low (*I*^2^ = 0%). The test for overall effect yielded *z* = 4.82 (*P* < 0.0001). The between-study variance was *τ*^2^ = 0.0, and the prediction interval ranged from 1.16 to 2.05. The meta-analysis considered T as the reference allele.

#### *ESR2*_rs3020450 (NM_001291723.1 (*ESR2*):c.18509G>A)

For the rs3020450 polymorphism in the ESR2 gene, two studies were included. No significant associations were found using the random-effects model (OR = 0.94; 95% CI: 0.77–1.13) (Fig. 3F). An *I*^2^ of 0% indicates no observed heterogeneity. The test for overall effect yielded *z* = −0.69 (*P* = 0.4922). The between-study variance was *τ*^2^ = 0.0, and the prediction interval ranged from 0.27 to 3.19. The G allele was considered the reference in the meta-analysis.

#### *CYP1A1*_rs1048943 (NM_000499.4 (*CYP1A1*):c.1384A>G)

This polymorphism was considered in six studies, and a meta-analysis was conducted. No significant associations were observed using the random-effects model (OR = 1.46; 95% CI: 0.84–2.52) (Fig. 3G). High heterogeneity was observed among these studies (*I*^2^ = 84.8%). The test for overall effect yielded *z* = 1.35 (*P* = 0.1756). The between-study variance was *τ*^2^ = 0.3522, and the prediction interval ranged from 0.27 to 7.88. The A allele was considered the reference in the meta-analysis.

#### *CYP1A1*_rs4646903 (NM_000499.5 (*CYP1A1*):c.11229T>C)

A total of four studies evaluated the rs4646903 polymorphism, and these were included in the meta-analysis. No significant associations were found using the random-effects model (OR = 0.93; 95% CI: 0.69–1.27) (Fig. 3H). The heterogeneity was low (*I*^2^ = 39.3%). The test for overall effect yielded *z* = −0.44 (*P* = 0.6605). The between-study variance was *τ*^2^ = 0.0387, and the prediction interval ranged from 0.42 to 2.08. The T allele was considered the reference in the meta-analysis.

### Analysis based on dominant and recessive genetic models

Additional analyses were performed using dominant and recessive genetic models for each included SNP. Overall, the results did not reveal statistically significant associations in any of the tested models, with the marginal exception of SNP rs743572 (*CYP17A1*) under the dominant model, which showed a trend toward association (OR = 1.56; 95% CI: 1.00–2.45; *P* = 0.050), despite moderate heterogeneity (*I*^2^ = 55.6%). Another potentially relevant finding was observed for SNP rs762551 (*CYP1A2*) under the dominant model, with an OR of 2.22 (95% CI: 1.18–4.18; *P* = 0.053), though its interpretation is limited due to high heterogeneity (*I*^2^ = 72.5%). All other models yielded *P*-values above 0.05 and generally showed low to moderate heterogeneity, as detailed in Supplementary Appendix D. These findings complement the main analyses by offering broader insight into possible inheritance patterns, although they do not represent the central results of this meta-analysis.

### Certainty of evidence analysis

The certainty of the evidence in this systematic review, according to GRADE criteria, was considered high for the Val158Met polymorphism in the *COMT* gene, Ile462Val in the *CYP1A1* gene, Ile105Val in the *GSTP1* gene, and A2 in the *CYP17* gene; moderate for the Val432Leu polymorphism in the *CYP1B1* gene, rs3020450, and T6235C Mspl; and very low for the CYP1A2*1F polymorphism (Table 2). Therefore, it is unlikely that future research will change the confidence in the effect estimate for studies with high certainty, whereas it is likely that future research will have a significant impact on the confidence in the effect estimate for studies with moderate certainty.

**Table 2:** Certainty of evidence analysis of the studies included in the meta-analysis.

No of studies	Certainty assessment						Effect		Certainty
	Study design	Risk of bias	Inconsistency	Indirect evidence	Imprecision	Other considerations	No. of events	No. of individuals	
***COMT*** ** Val158Met**									
6	Observational	Not serious	Not serious	Not serious	Not serious	None	1,445 cases	3,849 controls	⨁⨁⨁⨁ High
***A4889G CYP1A1*** ** Ile462Val**									
7	Observational	Not serious	Not serious	Not serious	Not serious	None	1,538 cases	4,133 controls	⨁⨁⨁⨁ High
***CYP1B1*** ** Val432Leu**									
5	Observational	Not serious	Serious (a)	Not serious	Not serious	None	1,566 cases	3,873 controls	⨁⨁⨁◯ Moderate
***GSTP1*** ** Ile105Val**									
3	Observational	Not serious	Not serious	Not serious	Not serious	None	418 cases	2,323 controls	⨁⨁⨁⨁ High
***T6235C*** ** mspl**									
5	Observational	Not serious	Not serious	Not serious	Serious (b)	None	1,423 cases	2,054 controls	⨁⨁⨁◯ Moderate
CYP1A2*1F									
3	Observational	Not serious	Very serious (a)	Not serious	Very serious (b)	None	756 cases	976 controls	⨁◯◯◯ Very low
***CYP17 A2***									
5	Observational	Not serious	Not serious	Not serious	Not serious	None	1,208 cases	1,766 controls	⨁⨁⨁⨁ High
**rs3020450**									
2	Observational	Not serious	Not serious	Not serious	Serious (b)	None	990 cases	1,506 controls	⨁⨁⨁◯ Moderate

Note: (a) heterogeneity of the meta-analysis for case-control studies was high (>60%); (b) does not present a narrow confidence interval; (c) small sample size. (*) specific gene variant.

## Discussion

We identified potential associations between ovarian cancer risk and the polymorphisms rs743572 (NM_000102.4(*CYP17A1*):c.−34T>C), rs4680 (NM_000754.4(*COMT*):c.472G>A (p.Val158Met)), and rs1695 (NM_000852.3:(*GSTP1*):c.313A>G (p.Ile105Val)). Specifically, the C allele of rs743572 (OR = 1.54; 95% CI (1.29–1.84)) was associated with an increased risk of ovarian cancer. Conversely, the A allele of rs4680 (OR = 0.85; 95% CI (0.73–0.99)) and the G allele of rs1695 (OR = 0.69; 95% CI (0.51–0.92)) were associated with a protective effect.

The additional analyses based on dominant and recessive genetic models provided complementary evidence for some of the key SNPs identified in the allele-contrast model. For rs743572 (*CYP17A1*), the dominant model yielded a borderline significant association (OR = 1.56; 95% CI: 1.00–2.45; *P* = 0.05), reinforcing the increased risk observed for the C allele in the main analysis. Similarly, rs4680 (*COMT*) showed a protective trend under the dominant model (OR = 0.80), which, although not statistically significant, aligns with the protective effect associated with the A allele in the allele-contrast results. In contrast, the analyses for rs1695 (*GSTP1*) did not show a consistent direction of association in either the dominant or recessive models, suggesting that the protective effect observed for the G allele in the main analysis may not extend to those inheritance patterns.

Among the analyzed genes, rs743572, located in *CYP17A1*, plays a role in steroid and sex hormone biosynthesis (Sohi & Singh 2018, Sun *et al.* 2018). While rarely studied in ovarian cancer, this polymorphism has been linked to phenotypic traits such as body height (Yengo *et al.* 2022). In contrast, rs4680 and rs1695, located in *COMT* and *GSTP1*, respectively, have not been linked to ovarian cancer in current GWAS databases but have been associated with other conditions, including chronic kidney disease (Schlosser *et al.* 2020, Surapaneni *et al.* 2022).

Despite these findings, our study faced several methodological limitations. The absence of ethnicity-specific data prevented subgroup analyses by ethnicity. Furthermore, the limited availability of studies on additional estrogen-related genes restricted the overall scope of this meta-analysis. For certain SNPs, only two or three eligible studies were available, which limited the precision of the between-study variance (Tau) estimates. Furthermore, the influence of environmental, nutritional, and psychological factors on ovarian cancer risk could not be evaluated, as these variables were not independently reported in the included studies. In addition, the lack of access to individual participant data across studies prevented us from performing adjusted analyses to control for potential confounding variables such as age, ethnicity, or hormonal exposure.

Significant publication bias was also observed for the rs762551 SNP (*CYP1A2* gene), as indicated by Egger’s regression test (*P* = 0.0021), suggesting that studies with negative or inconclusive results may be underrepresented, potentially affecting the reliability of the findings for this variant. Another noteworthy limitation is the absence of studies involving populations from North Africa and sub-Saharan Africa, which restricts the generalizability of our findings. In addition, due to limited data availability, it was not possible to perform subgroup analyses by ethnicity or cancer subtype, nor to conduct meta-regressions.

Our findings highlight the potential of these polymorphisms as targets for future research. More comprehensive case–control studies, as well as large-scale genome-wide association studies (GWAS), are essential to further investigate these associations. Such research could deepen our understanding of the molecular mechanisms underlying ovarian cancer and contribute to the identification of biomarkers for early diagnosis and personalized treatment.

## Conclusion

We conclude that certain polymorphisms in estrogen-related genes may be associated with either increased susceptibility (rs743572) or protection (rs4680 and rs1695) against ovarian cancer. However, the current body of the scientific literature remains limited in scope and power, which restricts the confirmation of these associations with high certainty. The evidence from this meta-analysis indicates that these polymorphisms are promising candidates for further investigation in future epidemiological and molecular studies.

## Supplementary materials



















## Declaration of interest

The authors declare that there is no conflict of interest that could be perceived as prejudicing the impartiality of the work reported.

## Funding

This study was financed in part by the Coordenação de Aperfeiçoamento de Pessoal de nível Superior- Brasil (CAPES) – Finance Code 001, through scholarships granted to the Federal University of Triângulo Mineiro.

## Author contribution statement

MPA, MOG, LFA, and LFSM contributed to the conceptualization, data curation, formal analysis, investigation, methodology, project administration, validation, and visualization. LFA and MOG performed the statistical analysis and the analysis of the certainty of the evidence. MPA was responsible for writing the original draft. APE contributed to project administration, supervision, and writing review and editing. All authors read and approved the final manuscript.

## References

[bib1] Abaoğlu İY, Yılmaz SG, Akdeniz FT, et al. 2001 Investigation of catechol-O-methyltransferase (COMT) gene Val158Met polymorphism in ovarian cancer. J Turk Ger Gynecol Assoc 22 42–46. (10.4274/jtgga.galenos.2020.2020.0091) PMC7944234 33389924

[bib3] Cavalieri EL & Rogan EG 2016 Depurinating estrogen-DNA adducts, generators of cancer initiation: their minimization leads to cancer prevention. Clin Transl Med 5 e12. (10.1186/s40169-016-0088-3) PMC4792821 26979321

[bib4] Da Silva AC, Jammal MP, Crispim PCA, et al. 2020 The role of stroma in ovarian cancer. Immunol Investig 49 406–424. (10.1080/08820139.2019.1658770) 32264761

[bib5] Delort L, Chalabi N, Satih S, et al. 2008 Association between genetic polymorphisms and ovarian cancer risk. Anticancer Res 28 3079–3081. 19031960

[bib6] Fasching PA, Gayther S, Pearce L, et al. 2009 Role of genetic polymorphisms and ovarian cancer susceptibility. Mol Oncol 3 171–181. (10.1016/j.molonc.2009.01.008) 19383379 PMC5527888

[bib7] Garner E, Stokes E, Berkowitz R, et al. 2002 Polymorphisms of the estrogen-metabolizing genes CYP17 and catechol-O-methyltransferase and risk of epithelial ovarian cancer. Cancer Res 62 3058–3062. 12036914

[bib8] Goodman MT, Lavigne JA, Hengstler JG, et al. 2000 Catechol-O-methyltransferase polymorphism is not associated with ovarian cancer risk. Cancer Epidemiol Biomarkers Prev 9 1373–1376. 11142424

[bib9] Goodman MT, McDuffie K, Kolonel LN, et al. 2001 Case-control study of ovarian cancer and polymorphisms in genes involved in catecholestrogen formation and metabolism. Cancer Epidemiol Biomarkers Prev 10 209-216. 11303589

[bib10] Goodman MT, Lurie G, Thompson PJ, et al. 2008 Association of two common single-nucleotide polymorphisms in the CYP19A1 locus and ovarian cancer risk. Endocr Relat Cancer 15 1055–1060. (10.1677/erc-08-0104) 18667686 PMC2663409

[bib11] Gulyaeva LF, et al. 2008 Comparative analysis of SNP in estrogen-metabolizing enzymes for ovarian, endometrial, and breast cancers in Novosibirsk, Russia. Adv Exp Med Biol 617 359–366. (10.1007/978-0-387-69080-3_34) 18497059

[bib44] Heubner M, Wimberger P, Riemann K, et al. 2010 The CYP1A1 Ile462Val polymorphism and platinum resistance of epithelial ovarian neoplasms. Oncol Res 18 343–347. (10.3727/096504010x12626118079903) 20377136

[bib36] Holt SK, Rossing MA, Malone EK, et al. 2007 Ovarian cancer risk and polymorphisms involved in estrogen catabolism. Cancer Epidemiol Biomarkers Prev 16 481–489. (10.1158/1055-9965.epi-06-0831) 17372243

[bib38] Kumar GG, Paul SFD, Molia C, et al. 2022 The association between CYP17A1, CYP19A1, and HSD17B1 gene polymorphisms of estrogen synthesis pathway and ovarian cancer predisposition. Meta Gene 31 100985. (10.1016/j.mgene.2021.100985)

[bib43] Leite DB 2009 Análise de polimorfismos nos genes CYP1A1, CYP17, COMT, GSTM1, receptor de estrogênios e progesterona em mulheres com carcinoma de ovário. Doctoral thesis. São Paulo, Brazil: Universidade Federal de São Paulo (UNIFESP). (http://repositorio.unifesp.br/handle/11600/8921)

[bib37] Lurie G, Wilkens LR, Thompson PJ, et al. 2009 Genetic polymorphisms in the estrogen receptor beta (ESR2) gene and the risk of epithelial ovarian carcinoma. Cancer Causes Control 20 47–55. (10.1007/s10552-008-9216-8) 18704709 PMC2663411

[bib42] Matei MC, Negură L, Liliac L, et al. 2012 Validation of PCR-RFLP techniques for the evaluation of codon 72 of p53 and CYP1A1 gene’s polymorphisms in relation with ovarian cancer in a Romanian population. Romanian Journal of Morphology and Embryology 22 42–46. (10.4274/jtgga.galenos.2020.2020.0091) 22395499

[bib12] Mikhailova ON, Gulyaeva LF, Prudnikov AV, et al. 2006 Estrogen-metabolizing gene polymorphisms in the assessment of female hormone-dependent cancer risk. Pharmacogenomics J 6 189–193. (10.1038/sj.tpj.6500365) 16402077

[bib14] Oliveira C, Lourenço GJ, Sagarra RAM, et al. 2012 Polymorphisms of glutathione S-transferase mu 1 (GSTM1), theta 1 (GSTT1), and pi 1 (GSTP1) genes and epithelial ovarian cancer risk. Dis Markers 33 155–159. (10.1155/2012/497692) 22960333 PMC3810705

[bib15] Page MJ, McKenzie JE, Bossuyt PM, et al. 2021 The PRISMA 2020 statement: an updated guideline for reporting systematic reviews. BMJ 372 n71. (10.1136/bmj.n71) 33782057 PMC8005924

[bib16] Pemmaraju S, Amidyala L, Vottery R, et al. 2018 Association of ER-α gene PvuII polymorphism with ovarian cancer. Cancer Treat Res Commun 14 13–16. (10.1016/j.ctarc.2017.11.001) 30104002

[bib19] Russo J & Russo IH 2006 The role of estrogen in the initiation of breast cancer. J Steroid Biochem Mol Biol 102 89–96. (10.1016/j.jsbmb.2006.09.004) 17113977 PMC1832080

[bib20] Dos Santos EVW, Alves LNR & Louro ID 2017 Steroid metabolism gene polymorphisms and their implications on breast and ovarian cancer prognosis. Genet Mol Res 16. (10.4238/gmr16039691) 28692125

[bib21] Schlosser P, Li Y, Sekula P, et al. 2020 Genetic studies of urinary metabolites illuminate mechanisms of detoxification and excretion in humans. Nat Genet 52 167–176. (10.1038/s41588-019-0567-8) 31959995 PMC7484970

[bib22] Schünemann HJ, Oxman AD, Brozek J, et al. 2008 Grading quality of evidence and strength of recommendations for diagnostic tests and strategies. BMJ 336 1106–1110. (10.1136/bmj.39500.677199.ae) 18483053 PMC2386626

[bib23] Schüler S, Lattrich C, Skrzypczak M, et al. 2014 Polymorphisms in the promoter region of ESR2 gene and susceptibility to ovarian cancer. Gene 546 283–287. (10.1016/j.gene.2014.05.066) 24881814

[bib24] Sellers TA, Schildkraut JM, Pankratz VS, et al. 2005 Estrogen bioactivation, genetic polymorphisms, and ovarian cancer. Cancer Epidemiol Biomarkers Prev 14 2536–2543. (10.1158/1055-9965.epi-05-0142) 16284375

[bib25] Seremak-Mrozikiewicz A, Drews K, Semczuk A, et al. 2005 CYP1A1 alleles in female genital cancers in the Polish population. Eur J Obstet Gynecol Reprod Biol 118 246–250. (10.1016/j.ejogrb.2004.06.036) 15653213

[bib26] Sohi N & Singh A 2018 Single nucleotide polymorphisms: identification and association with breast cancer using biocomputing approach. In 2018 Second International Conference on Intelligent Computing and Control Systems (ICICCS)*.* Piscataway, NJ, USA: IEEE, 2018. (10.1109/ICCONS.2018.8663085)

[bib27] Soltész L, Lukács J, Penyige J, et al. 2019 Determination of miR-193b rs30236 single nucleotide polymorphism in ovarian cancer patients. Eur J Gynaecol Oncol 40 547–550. (10.12892/ejgo4514.2019)

[bib28] Sugawara K, Nomura H, Sakuragi N, et al. 2003 CYP1A1 polymorphism and risk of gynecological malignancy in Japan. Int J Gynecol Cancer 13 785–790. (10.1136/ijgc-00009577-200311000-00009) 14675315

[bib29] Sun J, Zhang H, Gao M, et al. 2018 Association between CYP17 T-34C rs743572 and breast cancer risk. Oncotarget 9 4200–4213. (10.18632/oncotarget.23688) 29423115 PMC5790532

[bib30] Surapaneni A, Schlosser P, Zhou L, et al. 2022 Identification of 969 protein quantitative trait loci in an African American population with kidney disease attributed to hypertension. Kidney Int 102 1167–1177. (10.1016/j.kint.2022.07.005) 35870639 PMC12997408

[bib31] Viechtbauer W 2010 Conducting meta-analyses in R with the metafor package. J Stat Software 36 1–48. (10.18637/jss.v036.i03)

[bib32] Wu HJ, Sekine M, Kashima K, et al. 2005 Mutational analysis of the estrogen receptor-α gene in familial ovarian cancer. J Obstet Gynaecol Res 31 375–383. (10.1111/j.1447-0756.2005.00305.x) 16176503

[bib33] Yazici H, Tigli H, Kadehci Z, et al. 2006 Are CYP17 genotypes a biomarker for ovarian cancer in patients with cancer history in their family? Oncol Res 16 43–47. (10.3727/000000006783981279) 16783967

[bib34] Yengo L, Vedantam S, Marouli E, et al. 2022 A saturated map of common genetic variants associated with human height. Nature 610 704–712. (10.1038/s41586-022-05275-y) 36224396 PMC9605867

[bib35] Zahid M, Beseler CL, Hall JB, et al. 2014 Unbalanced estrogen metabolism in ovarian cancer. Int J Cancer 134 2414–2423. (10.1002/ijc.28565) 24170413 PMC3949171

