# Supplementary material for: Impact of single nucleotide variants in estrogen genes on ovarian cancer risk: a systematic review and meta-analysis
Source: Endocr Oncol. 2025 Aug 27;5(1):e250007. doi: 10.1530/EO-25-0007 (PMC12558086; doi:10.1530/EO-25-0007)
Supplement: Supplementary file 1 [file appendixA.pdf]

Appendix A - Search Strategies for each database

Data base	Search strategy
Embase	estrogen'/exp OR 'alpha estrogen' OR 'alpha oestrogen' OR 'beta estrogen' OR 'beta oestrogen' OR 'estrogen uptake' OR 'estrogene' OR 'estrogenic agent' OR 'estrogenic hormone' OR 'estrogenic steroids, alkylated' OR 'estrogens' OR 'estrogens, non steroidal' OR 'estrogens, non-steroidal' OR 'kober chromogen' OR 'oestrogen' OR 'oestrogen uptake' OR 'oestrogene' OR 'oestrogenic agent' OR 'oestrogenic hormone' OR 'oestrogenic steroids, alkylated' OR 'oestrogens' OR 'oestrogens, non steroidal' OR 'oestrogens, non-steroidal' OR 'estrogen' AND 'genetic polymorphism'/exp OR 'polymorphism (genetics)' OR 'polymorphism, genetic' OR 'genetic polymorphism' OR 'single nucleotide polymorphism'/exp OR 'polymorphism, single nucleotide' OR 'single nucleotide variant' OR 'single nucleotide variation' OR 'single nucleotide polymorphism' AND 'ovary tumor'/exp OR 'neoplasm of the ovary' OR 'neoplasms of the ovary' OR 'neoplastic ovarian' OR 'neoplastic ovaries' OR 'neoplastic ovary' OR 'ovarian neoplasia' OR 'ovarian neoplasm' OR 'ovarian neoplasms' OR 'ovarian tumor' OR 'ovarian tumorigenesis' OR 'ovarian tumour' OR 'ovarium tumor' OR 'ovarium tumour' OR 'ovary neoplasm' OR 'ovary tumor treatment' OR 'ovary tumorigenesis' OR 'ovary tumour' OR 'ovary tumour treatment' OR 'tumor of the ovary' OR 'tumors of the ovary' OR 'tumour of the ovary' OR 'tumours of the ovary' OR 'ovary tumor'
Lilacs	mh:Estrogênios OR Estrogênios OR (Efeito Estrogênio) OR Estrogênio Estrógenos OR Estrogens OR (Agents, Estrogenic) OR (Agonists, Estrogen Receptor) OR (Compounds, Estrogenic) OR (Effects, Estrogen) OR (Effects, Estrogenic) OR Estrogen OR (Estrogen Effect) OR (Estrogen Effects) OR (Estrogen Receptor Agonists) OR (Estrogenic Agents) OR (Estrogenic Compounds) OR (Estrogenic Effect) OR (Estrogenic Effects) OR (Receptor Agonists, Estrogen) OR Estrógenos OR (Efecto Estrógeno) OR Estrógeno OR mh:D27.505.696.399.472.277$ AND mh:"Polimorfismo Genético" OR (Polimorfismo Genético) OR (Polimorfismo (Genética)) OR (Polimorfismo do Gene) OR (Polymorphism, Genetic) OR (Gene Polymorphism) OR (Gene Polymorphisms) OR (Genetic Polymorphism) OR (Genetic Polymorphisms) OR (Polymorphism (Genetics)) OR (Polymorphism, Gene) OR (Polymorphisms (Genetics)) OR (Polymorphisms, Gene) OR (Polymorphisms, Genetic) OR (Polimorfismo Genético) OR (Polimorfismo Genética) OR mh:G05.365.795$ OR mh:"Polimorfismo de Nucleotídeo Único" OR (Polimorfismo de Nucleotídeo Único) OR (Polimorfismo de Um Único Nucleotídeo) OR (SNPs) OR (Polymorphism, Single Nucleotide) OR (Nucleotide Polymorphism, Single) OR (Nucleotide Polymorphisms, Single) OR (Polymorphisms, Single Nucleotide) OR (SNPs) OR (Single Nucleotide Polymorphism) OR (Single Nucleotide Polymorphisms) OR (Polimorfismo de Nucleótido Simple) OR (PNS) OR (PSN) OR (PSNS) OR (SNPs) OR mh:G05.365.795.598$ AND mh:"Neoplasias Ovarianas" OR (Neoplasias Ovarianas) OR (Câncer Ovariano) OR (Câncer de Ovário) OR (Polimorfismo (Genética)) OR (Polimorfismo do Gene) OR (Ovarian Neoplasms) OR (Cancer of Ovary) OR (Cancer of the Ovary) OR (Cancer, Ovarian) OR (Cancer, Ovary) OR (Cancers, Ovarian) OR (Cancers, Ovary) OR (Neoplasm, Ovarian) OR (Neoplasm, Ovary) OR (Neoplasms, Ovarian) OR (Neoplasms, Ovary) OR (Ovarian Cancer) OR (Ovarian Cancers) OR (Ovarian Neoplasm) OR (Ovary Cancer) OR (Ovary Cancers) OR (Ovary Neoplasm) OR (Ovary Neoplasms) OR (Neoplasias Ováricas) OR (Cáncer Ovárico) OR (Cáncer de Ovario) OR mh:C04.588.322.455$ OR mh:C12.050.351.500.056.630.705$ OR mh:C12.050.351.937.418.685$ OR mh:C12.100.250.056.630.705$ OR mh:C12.900.418.685$ OR mh:C19.344.410$ OR mh:C19.391.630.705$

Appendix A - Continuation

Data base	Search strategy
Pubmed	"Estrogens"[Mesh] OR Estrogens OR (Estrogenic Compounds) OR (Compounds, Estrogenic) OR (Estrogenic Agents) OR (Agents, Estrogenic) OR Estrogen OR (Estrogen Receptor Agonists) OR (Agonists, Estrogen Receptor) OR (Receptor Agonists, Estrogen) OR (Estrogen Effect) OR (Estrogenic Effect) OR (Estrogenic Effects) OR (Effects, Estrogenic) OR (Estrogen Effects) OR (Effects, Estrogen) AND "Polymorphism, Genetic"[Mesh] OR (Polymorphism, Genetic) OR (Polymorphisms, Genetic) OR (Genetic Polymorphism) OR (Genetic Polymorphisms) OR (Gene Polymorphism) OR (Gene Polymorphisms) OR (Polymorphism, Gene) OR (Polymorphisms, Gene) OR (Polymorphism (Genetics)) OR (Polymorphisms (Genetics)) OR "Polymorphism, Single Nucleotide"[Mesh] OR (Polymorphism, Single Nucleotide) OR (Nucleotide Polymorphism, Single) OR (Nucleotide Polymorphisms, Single) OR (Polymorphisms, Single Nucleotide) OR (Single Nucleotide Polymorphisms) OR (SNPs) AND "Ovarian Neoplasms"[Mesh] OR (Ovarian Neoplasms) OR (Neoplasm, Ovarian) OR (Ovarian Neoplasm) OR (Ovary Neoplasms) OR (Neoplasm, Ovary) OR (Neoplasms, Ovary) OR (Ovary Neoplasm) OR (Neoplasms, Ovarian) OR (Ovary Cancer) OR (Cancer, Ovary) OR (Cancers, Ovary) OR (Ovary Cancers) OR (Ovarian Cancer) OR (Cancer, Ovarian) OR (Cancers, Ovarian) OR (Ovarian Cancers) OR (Cancer of Ovary) OR (Cancer of the Ovary)
Scopus	Estrogens OR "Estrogenic Compounds" OR "Compounds, Estrogenic" OR "Estrogenic Agents" OR "Agents, Estrogenic" OR Estrogen OR "Estrogen Receptor Agonists" OR "Agonists, Estrogen Receptor" OR "Receptor Agonists, Estrogen" OR "Estrogen Effect" OR "Estrogenic Effect" OR "Estrogenic Effects" OR "Effects, Estrogenic" OR "Estrogen Effects" OR "Effects, Estrogen" AND "Polymorphism, Genetic" OR "Polymorphisms, Genetic" OR "Genetic Polymorphism" OR "Genetic Polymorphisms" OR "Gene Polymorphism" OR "Gene Polymorphisms" OR "Polymorphism, Gene" OR "Polymorphisms, Gene" OR "Polymorphism (Genetics)" OR "Polymorphisms (Genetics)" OR "Polymorphism, Single Nucleotide" OR "Nucleotide Polymorphism, Single" OR "Nucleotide Polymorphisms, Single" OR "Polymorphisms, Single Nucleotide"OR "Single Nucleotide Polymorphisms" OR "SNPs" AND "Ovarian Neoplasms" OR "Neoplasm, Ovarian" OR "Ovarian Neoplasm" OR "Ovary Neoplasms" OR "Neoplasm, Ovary" OR "Neoplasms, Ovary" OR "Ovary Neoplasm" OR "Neoplasms, Ovarian" OR "Ovary Cancer" OR "Cancer, Ovary"OR "Cancers, Ovary" OR "Ovary Cancers" OR "Ovarian Cancer" OR "Cancer, Ovarian" OR "Cancers, Ovarian"OR "Ovarian Cancers" OR "Cancer of Ovary" OR "Cancer of the Ovary"
Web of Science	Estrogens OR "Estrogenic Compounds" OR "Compounds, Estrogenic" OR "Estrogenic Agents" OR "Agents, Estrogenic" OR Estrogen OR "Estrogen Receptor Agonists" OR "Agonists, Estrogen Receptor" OR "Receptor Agonists, Estrogen" OR "Estrogen Effect" OR "Estrogenic Effect" OR "Estrogenic Effects" OR "Effects, Estrogenic" OR "Estrogen Effects" OR "Effects, Estrogen" AND "Polymorphism, Genetic" OR "Polymorphisms, Genetic" OR "Genetic Polymorphism" OR "Genetic Polymorphisms" OR "Gene Polymorphism" OR "Gene Polymorphisms" OR "Polymorphism, Gene" OR "Polymorphisms, Gene" OR "Polymorphism (Genetics)" OR "Polymorphisms (Genetics)" OR "Polymorphism, Single Nucleotide" OR "Nucleotide Polymorphism, Single" OR "Nucleotide Polymorphisms, Single" OR "Polymorphisms, Single Nucleotide"OR "Single Nucleotide Polymorphisms" OR "SNPs" AND "Ovarian Neoplasms "OR "Neoplasm, Ovarian" OR "Ovarian Neoplasm" OR "Ovary Neoplasms" OR "Neoplasm, Ovary" OR "Neoplasms, Ovary" OR "Ovary Neoplasm" OR "Neoplasms, Ovarian" OR "Ovary Cancer" OR "Cancer, Ovary"OR "Cancers, Ovary" OR "Ovary Cancers" OR "Ovarian Cancer" OR "Cancer, Ovarian" OR "Cancers, Ovarian"OR "Ovarian Cancers" OR "Cancer of Ovary"

	OR "Cancer of the Ovary"

Appendix A - Continuation

Data base	Search strategy
Gray literature (Google Scholar)	(Estrogens AND "Polymorphism, Genetic" OR "Polymorphism, Single Nucleotide" AND "Ovarian Neoplasms")
