# Supplementary material for: Impact of single nucleotide variants in estrogen genes on ovarian cancer risk: a systematic review and meta-analysis
Source: Endocr Oncol. 2025 Aug 27;5(1):e250007. doi: 10.1530/EO-25-0007 (PMC12558086; doi:10.1530/EO-25-0007)
Supplement: Supplementary file 7 [file supplementary_materials1.pdf]

Appendix B - Excluded articles with their respective exclusion criteria

Author/ Year	Reasons for exclusion
ANDERSEN, T.I. *et al.* 1997	1
ASHTON, K.A. *et al.* 2006	1
BARTEL, F. *et al.* 2008	4
BOJESEN, S.E. *et al.* 2013	1
DOHERTY, J.A. *et al.* 2010	3
FENG, Y. et al. 2019	3
FOULKES, W.D. et al. 1993	1
HANSEN, L.L. et al. 2002	1
HUANG, X. *et al.* 2019	4
KAUFMAN, B. et al. 2011	2
KUTILIN, D.S. et al. 2021	1
LEE, J.H. et al. 1990	1
LURIE, G. *et al.* 2011	3
PEARCE, C.L. *et al.* 2008	3
PEARCE, C.L. *et al.* 2009	3
SANTOS, D.S.N.M. *et al.* 2010	1

Notes: 1- Does not answer the systematic review question; 2 - Wrong population; 3 - Study based on other works; 4 - Gene not related to estrogen.

REFERENCES

Anderson TI, Wooster R, Laake K, et al. Screening for ESR mutations in breast and ovarian cancer patients. *Hum Mutat*. 1997;9(6):531-536.

Ashton KA, Meldrum CJ, McPhillips ML, et al. The Association of the COMT V158M Polymorphism with Endometrial/Ovarian Cancer in HNPCC Families Adhering to the Amsterdam Criteria. *Hered Cancer Clin Pract*. 2006;4(2):94-102.

Bartel F, Jung J, Böhnke A, et al. Both germ line and somatic genetics of the p53 pathway affect ovarian cancer incidence and survival. *Clin Cancer Res*. 2008;14(1):89-96.

Bojesen SE, Pooley KA, Johnatty SE, et al. Multiple independent variants at the TERT locus are associated with telomere length and risks of breast and ovarian cancer. *Nat Genet*. 2013;45(4):371-384e3842.

Doherty JA, Rossing MA, Cushing-Haugen KL, et al. ESR1/SYNE1 polymorphism and invasive epithelial ovarian cancer risk: an Ovarian Cancer Association Consortium study. *Cancer Epidemiol Biomarkers Prev*. 2010;19(1):245-250.

Feng Y, Peng Z, Liu W, et al. Evaluation of the epidemiological and prognosis significance of ESR2 rs3020450 polymorphism in ovarian cancer. *Gene*. 2019;710:316-323.

Foulkes WD, Ragoussis J, Stamp GW, Allan GJ, Trowsdale J. Frequent loss of heterozygosity on chromosome 6 in human ovarian carcinoma. *Br J Cancer*. 1993;67(3):551-559.

Hansen LL, Jensen LL, Dimitrakakis C, et al. Allelic imbalance in selected chromosomal regions in ovarian cancer. *Cancer Genet Cytogenet*. 2002;139(1):1-8.

Huang X, Shen C, Zhang Y, et al. Associations between *TAB2* Gene Polymorphisms and Epithelial Ovarian Cancer in a Chinese Population. *Dis Markers*. 2019;2019:8012979.

Kaufman B, Laitman Y, Ziv E, et al. The CYP17A1 -34T > C polymorphism and breast cancer risk in BRCA1 and BRCA2 mutation carriers. *Breast Cancer Res Treat*. 2011;126(2):521-527.

Kutilin DS, Tsandekova MR, Porkhanova NV. Features of the Copy Number Variation of Certain Genes in Tumor Cells in Patients with Serous Ovarian Adenocarcinoma. *Bull Exp Biol Med*. 2021;170(3):332-339.

Lee JH, Kavanagh JJ, Wildrick DM, Wharton JT, Blick M. Frequent loss of heterozygosity on chromosomes 6q, 11, and 17 in human ovarian carcinomas. *Cancer Res*. 1990;50(9):2724-2728.

Lurie G, Wilkens LR, Thompson PJ, et al. Estrogen receptor beta rs1271572 polymorphism and invasive ovarian carcinoma risk: pooled analysis within the Ovarian Cancer Association Consortium. *PLoS One*. 2011;6(6):e20703.

Leigh Pearce C, Near AM, Butler JL, et al. Comprehensive evaluation of ESR2 variation and ovarian cancer risk. *Cancer Epidemiol Biomarkers Prev*. 2008;17(2):393-396.

Pearce CL, Near AM, Van Den Berg DJ, et al. Validating genetic risk associations for ovarian cancer through the international Ovarian Cancer Association Consortium [published correction appears in Br J Cancer. 2009 Nov 17;101(10):1805. Cunningham, J C [corrected to Cunningham, J M]]. *Br J Cancer*. 2009;100(2):412-420.

Santos DSNM, Assis J, Gomes M, Rodrigues A.C.F., Oliveira J, Pereira D, Medeiros R. CYP3A4*1B (rs2740574) Gene polymorphism has prognostic value in ovarian cancer. *Ann Oncol.* 2010;21(8):viii310.
