# Supplementary material for: Impact of single nucleotide variants in estrogen genes on ovarian cancer risk: a systematic review and meta-analysis
Source: Endocr Oncol. 2025 Aug 27;5(1):e250007. doi: 10.1530/EO-25-0007 (PMC12558086; doi:10.1530/EO-25-0007)
Supplement: Supplementary file 9 [file supplementary_materials3.pdf]

Appendix C - Supplementary Material on Publication Bias Assessment

A - *COMT* (rs4680)

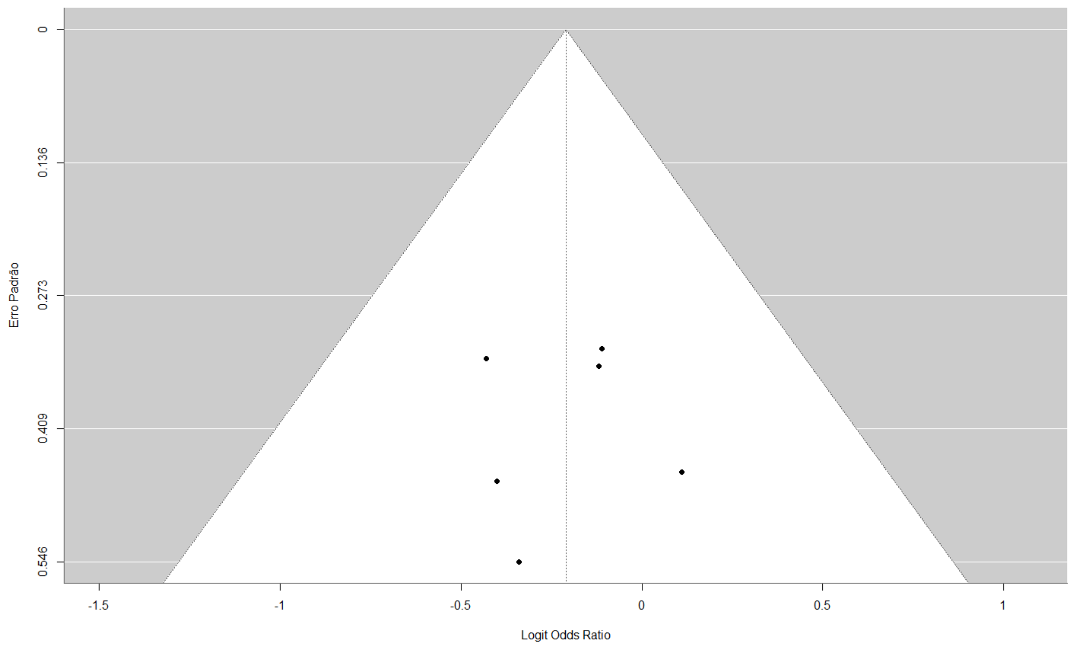


```
Regression Test for Funnel Plot Asymmetry

Model:     fixed-effects meta-regression model
Predictor: standard error

Test for Funnel Plot Asymmetry: z = -0.0571, p = 0.9545
Limit Estimate (as sei -> 0):   b = -0.1615 (CI: -1.8591, 1.5361)
```

B - *CYP1B1* (rs1056836)

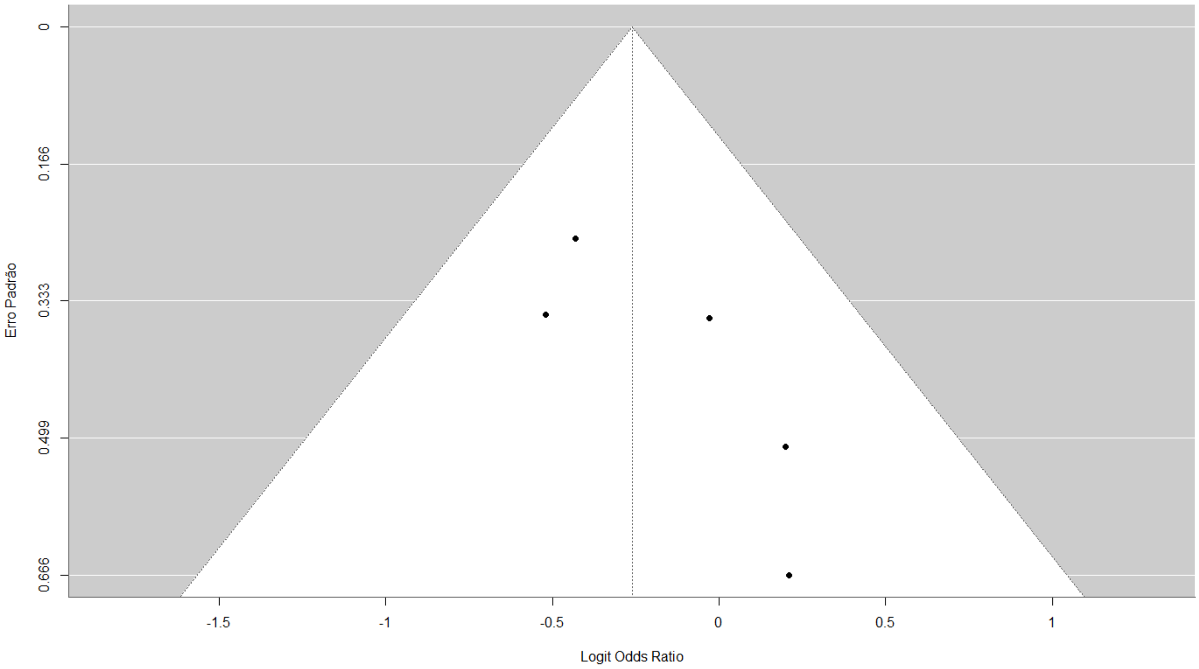


```
Regression Test for Funnel Plot Asymmetry

Model:     mixed-effects meta-regression model
Predictor: standard error

Test for Funnel Plot Asymmetry: z =  1.2839, p = 0.1992
Limit Estimate (as sei -> 0):   b = -0.9244 (CI: -1.9867, 0.1378)
```

C - *GSTP1* (rs1695)

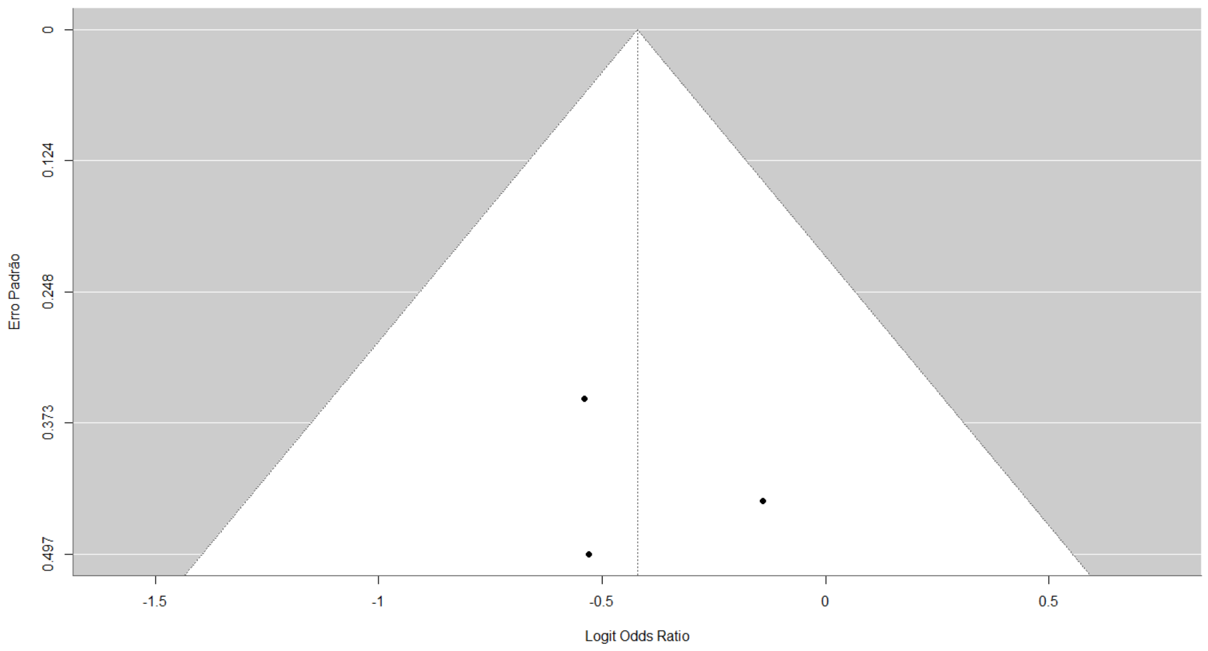


```
Regression Test for Funnel Plot Asymmetry

Model:     fixed-effects meta-regression model
Predictor: standard error

Test for Funnel Plot Asymmetry: z =  0.2768, p = 0.7820
Limit Estimate (as sei -> 0):   b = -0.8608 (CI: -4.0090, 2.2874)
```

D - *CYP1A2* (rs762551)

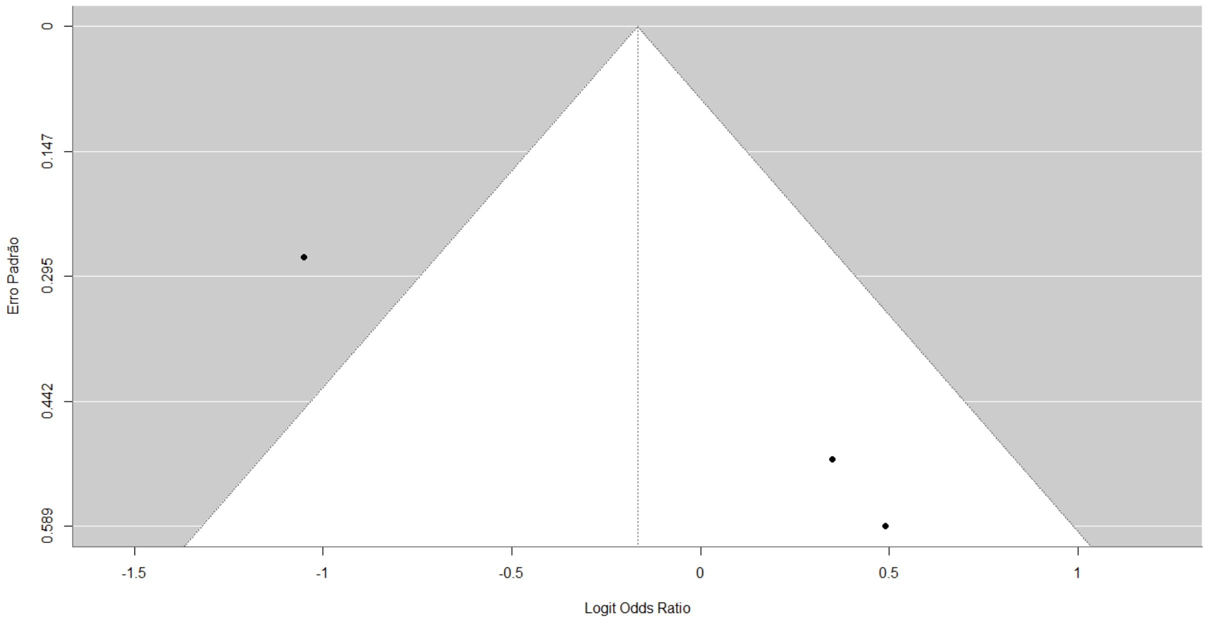


```
Regression Test for Funnel Plot Asymmetry

Model:     mixed-effects meta-regression model
Predictor: standard error

Test for Funnel Plot Asymmetry: z =  3.0784, p = 0.0021
Limit Estimate (as sei -> 0):   b = -2.4719 (CI: -3.7619, -1.1818)
```

E - *CYP19* (rs700519)

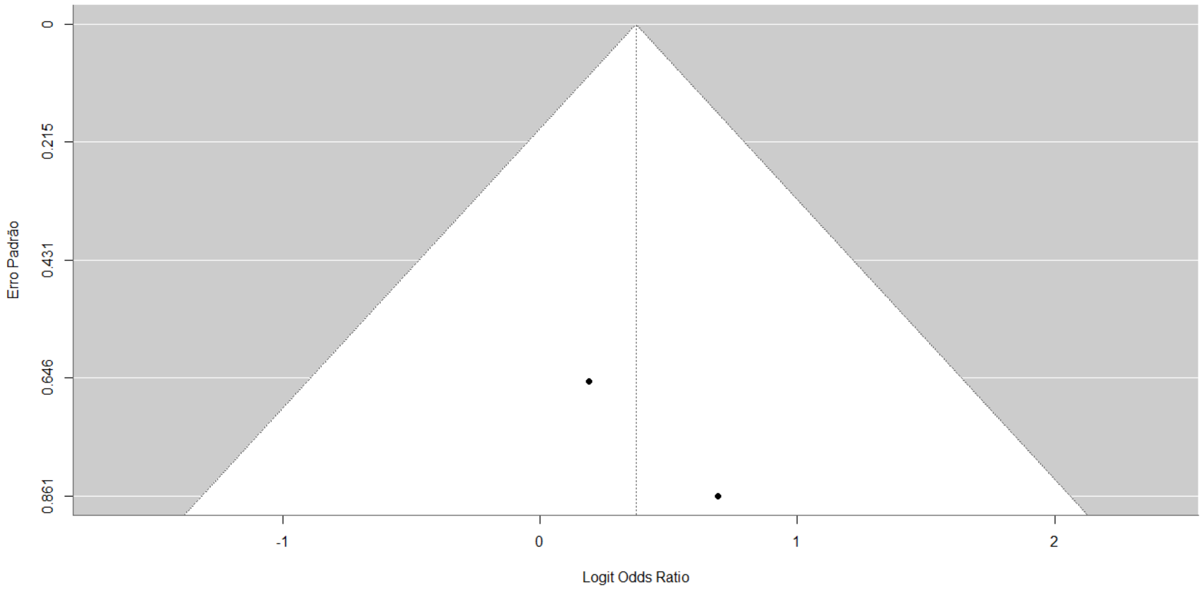


```
Regression Test for Funnel Plot Asymmetry

Model:     fixed-effects meta-regression model
Predictor: standard error

Test for Funnel Plot Asymmetry: z =  0.4626, p = 0.6436
Limit Estimate (as sei -> 0):   b = -1.3736 (CI: -8.8404, 6.0931)
```

F - *SULT1A1* (rs9282861)

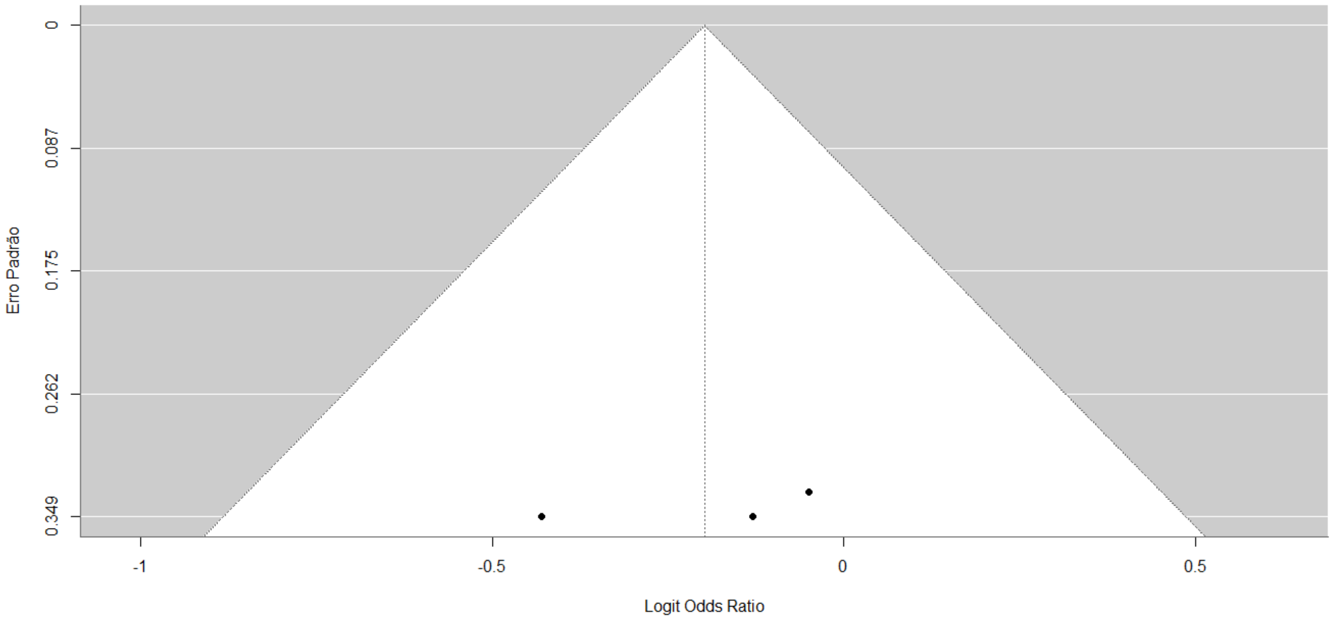


```
Regression Test for Funnel Plot Asymmetry

Model:     fixed-effects meta-regression model
Predictor: standard error

Test for Funnel Plot Asymmetry: z = -0.5562, p = 0.5781
Limit Estimate (as sei -> 0):   b =  4.2787 (CI: -11.5011, 20.0585)
```

G - *CYP17* (rs743572)

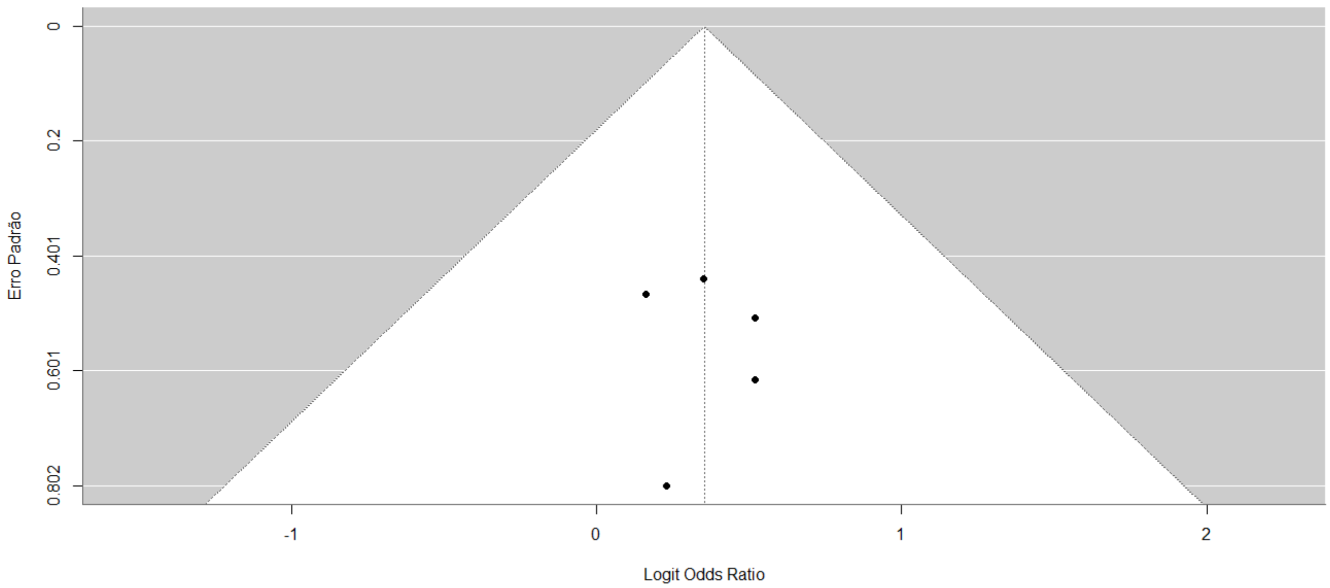


```
Regression Test for Funnel Plot Asymmetry

Model:     fixed-effects meta-regression model
Predictor: standard error

Test for Funnel Plot Asymmetry: z = 0.0661, p = 0.9473
Limit Estimate (as sei -> 0):   b = 0.2745 (CI: -2.0906, 2.6396)
```

H - *ESR1* (rs3020450)

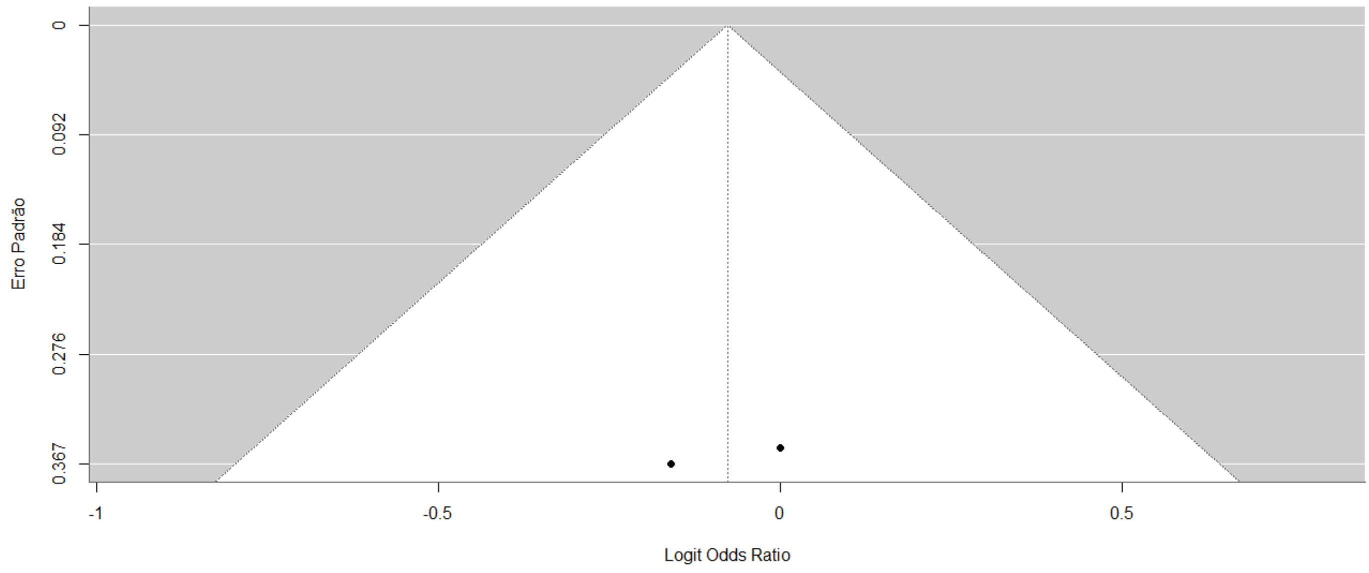


```
Regression Test for Funnel Plot Asymmetry

Model:     fixed-effects meta-regression model
Predictor: standard error

Test for Funnel Plot Asymmetry: z = -0.3138, p = 0.7537
Limit Estimate (as sei -> 0):   b =  4.0785 (CI: -21.8816, 30.0386)
```

I - *ESR2* (rs2234693)

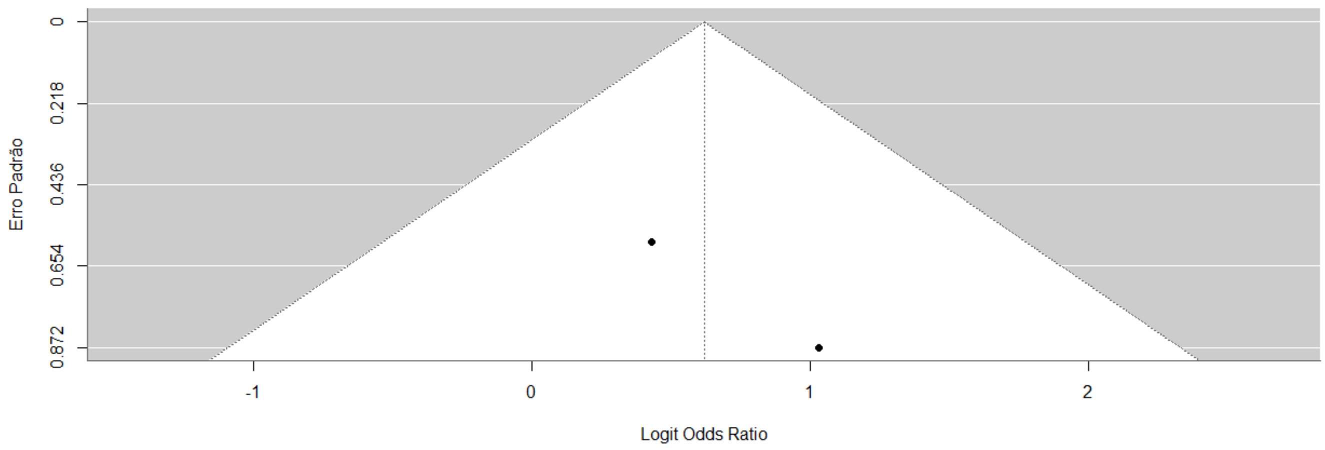


```
Regression Test for Funnel Plot Asymmetry

Model:     fixed-effects meta-regression model
Predictor: standard error

Test for Funnel Plot Asymmetry: z =  0.5698, p = 0.5688
Limit Estimate (as sei -> 0):   b = -0.8313 (CI: -5.9112, 4.2485)
```

J - *CYP1A1* (rs1048943)

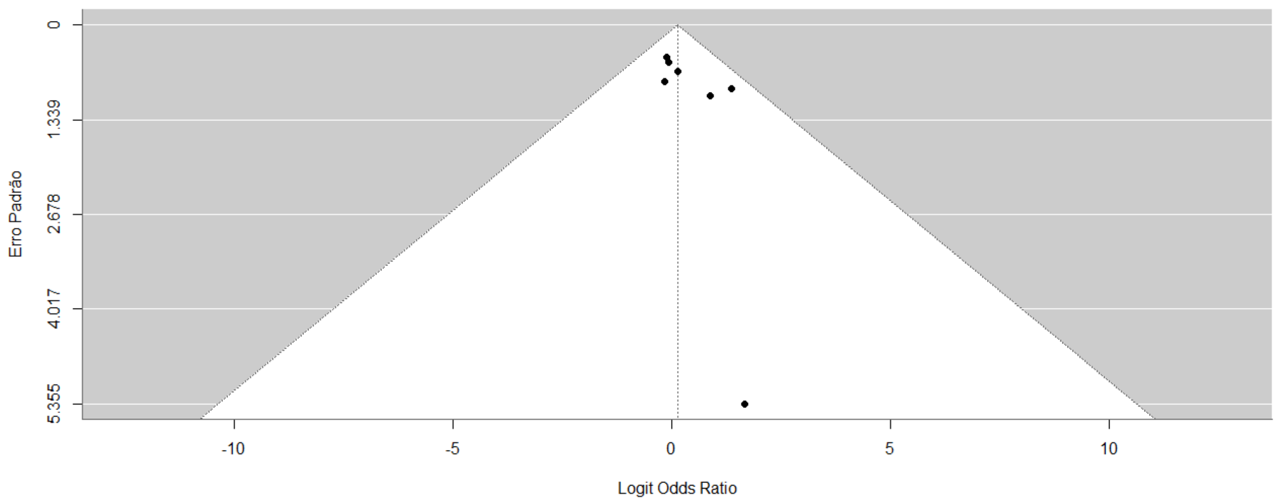


```
Regression Test for Funnel Plot Asymmetry

Model:     mixed-effects meta-regression model
Predictor: standard error

Test for Funnel Plot Asymmetry: z =  1.0215, p = 0.3070
Limit Estimate (as sei -> 0):   b = -0.4520 (CI: -1.6866, 0.7827)
```

L - *CYP1A1* (rs4646903)

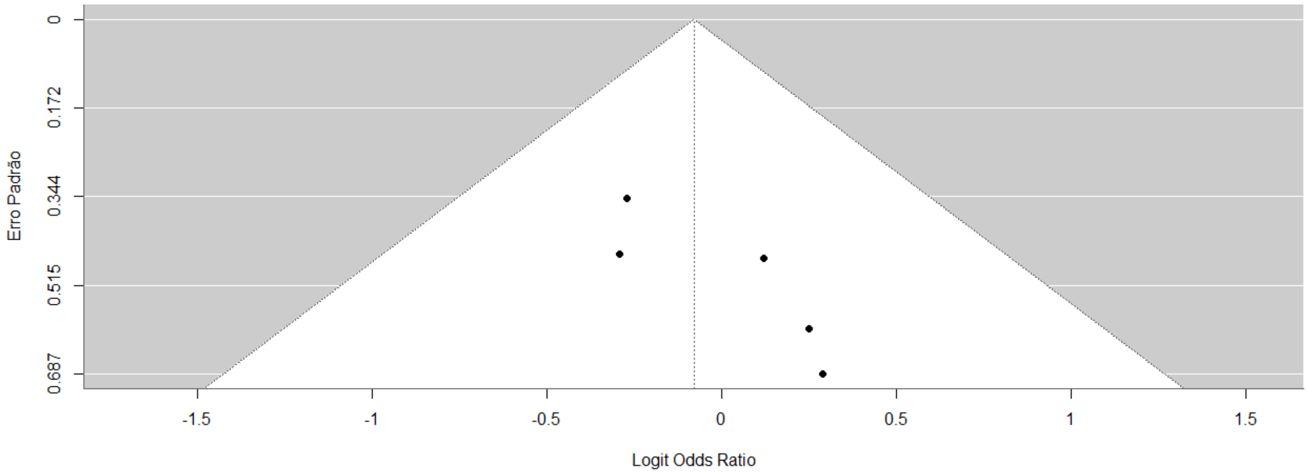


```
Regression Test for Funnel Plot Asymmetry

Model:     fixed-effects meta-regression model
Predictor: standard error

Test for Funnel Plot Asymmetry: z =  0.9487, p = 0.3428
Limit Estimate (as sei -> 0):   b = -0.9096 (CI: -2.6754, 0.8562)
```

REFERENCE

Peng, Z., Lv, X., Sun, Y., & Dai, S. Association of Interleukin-10-1082A/G Polymorphism with Idiopathic Recurrent Miscarriage: A Systematic Review and Meta-Analysis. Am J Reprod Immunol. 2016 Feb;75(2):162-71. doi: 10.1111/aji.12467.
